# Orthopedic Surgery

**Published:** 1902-04

**Authors:** Prescott LeBreton

**Affiliations:** Buffalo, N. Y.


### PROGRESS IN MEDICAL SCIENCE.

### Orthopedic Surgery.

CONDUCTED BY PRESCOTT LEBRETON, M. D., Buffalo, N. Y.

### REVIEW OF RECENT LITERATURE ON TUBERCULOUS DISEASE OF THE HIP-JOINT.

FIXATION, traction and protection are easily provided for knee and ankle joints, but the same treatment is procured with difficulty for hip joint, because of its ball and socket movement and because it forms the junction of trunk and lower limb. The solution of the problem as to what method or combination of methods yields the best results in hip disease has not yet been reached, although discussion has been continued for years past. Whitman (Orthopedic Surgery, 1901,) details the American, English and German methods of treatment and their advantages and disadvantages. The American method, socalled, after subsidence of acute symptoms, consists in applying the well-known traction splint, with its pelvic band, perineal straps, upright and foot piece, traction being made by rack and pinion and the moleskin plaster attached to the leg. When this apparatus is used as a walking brace, constant traction is not exerted, as the straps alternately relax and tighten when the weight of the body falls upon and leaves the brace in walking. Little actual traction is exerted ordinarily, although motion at the joint is restricted. Lovett's experiments show that the hip describes an arc of 35° of joint motion. Of course, if a high shoe is worn on the opposite foot and crutches are employed, traction is uninterrupted, but Davis, its inventor, and Taylor, who made it practicable, meant it for a walking brace.

Thomas, of Liverpool, in 1875, introduced his splint so extensively used in England, consisting of a flat piece of malleable iron, extending from the lower angle of the scapula to the middle of the calf, with chest, thigh and leg bands. This brace and the plaster-of-Paris spica, employed mostly by the Germans, afford fixation, but the Thomas splint is inefficient in antagonising lateral distortion, and the plaster-of-Paris is heavy, dirty and requires frequent renewals.

Whitman has experimented with cases with the idea of trying to combine in a brace all the advantages and none of the disadvantages of the other methods; in other words, to grant fixation, traction and protection without the production of flexion, which often occurs after the use of the traction brace, or lateral distortion, which occurs after the use of the Thomas splint. Whitman used first a spica of plaster-of-Paris under the ordinary traction brace. Then he exchanged the plaster for a short Thomas brace beneath the traction brace Then he combined the two by attaching a lateral thoracic bar to the pelvis band of the traction brace with metal band encircling the chest. This combination brace is now in general use at the Hospital for Ruptured and Crippled. It is to be noted that Ridlon at one time used a brace identical with this, and that Phelps has described a form of brace with a thoracic support, and with arrangements for lateral traction.

Dane, of Boston, (*New York Medical Journal*, August 24, 1901,) found the ordinary traction brace unsatisfactory, because the upper pelvic arm is short and grasps the pelvis very imperfectly, thus affording poor fixation. He followed Whitman's plan of the thoracic extension for two years and concluded that the grasp of the chest was even more unsatisfactory than the grasp of the pelvis. He decided that the thoracic arm served only as a lever by which all the motions of the trunk were transmitted directly to the hip joint, and that the compensating action of the lumbar spine, the "third hip joint," was only possible when left perfectly free and unhampered by any apparatus. He then modified his original brace which was similar to the ordinary traction splint, by adding a second posterior pelvic arm, carried as far down as possible over the sacrum, while the upper followed closely under the curve made by the crests of the ilia. A thick piece of leather was riveted firmly to the two arms, thus allowing a firmer grasp of the pelvis. The result showed an arc of motion of only six degrees.

Lovett (*New York Medical Journal*, August 24, 1901,) has made a strong plea against routinism in the treatment of hip disease. He believes that motion beyond the limit set by the reflex spasm is harmful, and is an advocate not only of traction, but strong and efficient traction, while fixation, although of great importance in acute cases with limited range of motion, is in general inferior to traction. For the treatment of severe cases allowing less than 20 degrees or 30 degrees of motion in flexion, Lovett has used with success a sole leather spica, reaching from the upper chest to the calf, outside of which is attached a skeleton long traction splint. With this treatment recumbency proved unnecessary. The splint was removed as seldom as possible. Cases allowing 25 degrees to 45 degrees of motion in flexion may be treated by Dane's splint with high shoe and crutches. The ordinary long traction splint should be limited to cases allowing well over 45 degrees of motion and should be used with high shoe and crutches.

Jouon (*These de Paris*, 1901,) quoted by Ostheimer (*Philadelphia Medical Journal*, February 1, 1902,) reports six cases of dislocation of the hip appearing spontaneously at the outset of hip disease. Hydrarthrosis is the predisposing cause and muscular effort the exciting cause. The dislocations were generally backward in type. The prognosis is excellent and treatment consists in reduction under anesthesia, a plaster-of-Paris spica and extension. La Guichaoua (*These de Paris*, 1901,) believes that the iliac variety is most frequent. When reduction is impossible, he advises osteotomy of the femur with the hope that ankylosis in good position may be the result.

